# Optimal design of dual air-gap closed-loop TMR current sensor based on minimum magnetic field uniformity coefficient

**DOI:** 10.1038/s41598-022-26971-9

**Published:** 2023-01-05

**Authors:** Jicheng Yu, Zhaozhi Long, Siyuan Liang, Changxi Yue, Xiaodong Yin, Feng Zhou

**Affiliations:** China Electric Power Research Institute, Wuhan, 430070 China

**Keywords:** Engineering, Electrical and electronic engineering

## Abstract

Advanced sensor technology provides accurate information for transparent monitoring and real-time control of the power grid. Tunnel magnetoresistance (TMR) elements with high sensitivity and linearity provide a new technical means for current measurement in medium-voltage DC power distribution systems. This paper proposes a dual air-gap closed-loop TMR current sensor and its optimal design method based on the magnetic field’s minimum uniformity coefficient. The dual air-gap structure reduces the measurement error caused by the eccentricity of the wire, and the theory and modelling of the minimum magnetic field uniformity coefficient optimise the key parameters, such as the inner radius of the magnetic core, the distance of the air-gap and the area size of the section side. Finally, a sensor prototype with a rated measurement current of ± 50 A was developed. The experiment results show that the relative error of the proposed TMR current sensor is less than 0.2% under the rated current. The conclusion can be drawn that the proposed sensor with the optimised design effectively improves the measurement accuracy.

## Introduction

Advanced sensor technology provides accurate information for the monitoring and controls of the power system. In recent years, with the development of power electronics devices, the distributed power sources and loads with power electronic elements, such as photovoltaics, battery storages and electric vehicle charging piles, have been increasingly connected with power distribution systems. As a result, a plenty of transient waveforms are injected to the grid, and this makes the measurement and sensing of the current more difficult. Higher requirements are put forward for the current sensors with accurate DC high current measurement capabilities, wide-frequency characteristics and inexpensive^1,2^.

Current sensors with magnetic sensor, such as Hall or tunnel magnetoresistance (TMR), are possible solutions. Hall-effect sensor has been around for decades and widely applied. However, Hall-effect sensor has inherent defects, such as weak sensitivity, low linearity but temperature sensitive^3,4^. The fourth-generation magnetic sensing element TMR has advanced characteristics in sensitivity, power consumption and temperature characters^5–7^. Current sensors with TMR elements is a new and better choice for complex waveform current measurement, but some technical issues need to be solved, such as the structure of the sensor, parameter settings, etc.

Ironless open-loop structure based TMR current sensors were the first development few years ago. Xu et al. designed a differential ultra-miniature magnetic sensor, which can measure the current of ± 150 A, and the experimental error is less than ± 2% in the temperature range of − 40 °C to 105 °C^2^. Shao et al. applied the TMR current sensor to insulated gate bipolar transistor (IGBT) overcurrent protection and proposed a ring array TMR current sensor to measure the IGBT current. The designed current sensor can detect the overcurrent of 120A within 604 ns^8^. However, the open-loop structure based TMR current sensor has two primary defects: First, the current sensor measurement range is limited by the linearity range of the TMR sensor element, therefor the rated current of this type of current sensor is limited within around a hundred amperes. Second, this type of current sensor is sensible to the change of temperature and also the current-carrying conductor eccentricity.

To increase the current measurement range and improve the temperature characteristics, scholars have integrated the zero-flux technology into the current measurement^9,10^. Yang proposed a closed-loop current sensor based on the zero-flux principle^11^, using a magnetic core and a feedback winding to form a closed-loop structure to improve the sensor sensitivity and vastly reduce errors caused by temperature and hysteresis. However, in practical applications, the current-carrying conductor is sometimes not at the centre of the magnetic circuit. The closed-loop current sensor is not very resistant to this eccentricity error^12^. Cheng et al. systematically analysed various characteristics of the magnetic core to study the influencing factors of the magnetic core of the closed-loop current sensor^13^. Aiming at the problem that the magnetic core may be saturated, Li proposed a closed-loop circuit without a magnetic core, which directly wound the solenoid composed of the feedback coil on the sensor element^14^. Roland et al. proposed a new coreless current sensor based on a circular magnetic field sensor array and applied the closed-loop principle to a circular array. However, this coreless structure is susceptible to external magnetic field interference. It is necessary to strictly ensure the uniformity of the coils wound on the annular array^15^, which is difficult to achieve in low-cost mass production. In addition, the existence of nearby interfering conductors and the placement of the sensor element will lead to changes in the magnetic induction intensity measured at the air-gap, which will also affect the sensor’s measurement accuracy^16^. The specific source of measurement error still needs to be analysed in depth, and an improved method should be determined for the error.

To solve the above problems, this paper proposes a dual air-gap closed-loop TMR current sensor and an optimal design method based on the uniformity coefficient of the magnetic field. “Design of dual air-gap closed-loop TMR current sensor” section analyses the structure and error source of dual air-gap closed-loop TMR current sensor and proposes the concept of minimum magnetic field uniformity coefficient. Based on this, in “Parameter optimisation based on the minimum magnetic field uniformity coefficient” section, the optimal design method of the sensor is proposed, and the critical parameters of the magnetic core are determined. In “Experiment results” section, the developed experimental prototype is tested.

## Design of dual air-gap closed-loop TMR current sensor

This paper proposes a dual air-gap closed-loop structure based TMR current sensor with iron core. The proposed TMR current sensor has better measurement characteristics with a low response time, a good linearity, low eccentric error and less influenced by the external magnetic interference.

### Structure and principle

**Figure 1:**
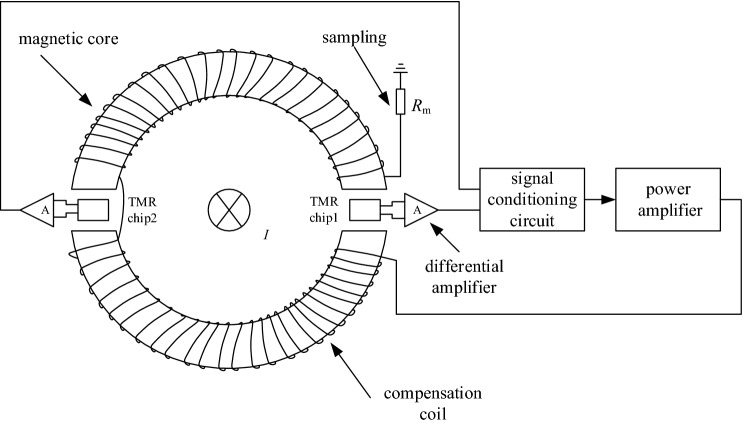
Structure of dual air-gap closed-loop TMR current sensor.

The designed dual air-gap TMR current sensor and its closed-loop feedback loop are shown in Fig. 1. The TMR elements work in the state of zero magnetic flux through the magnetic core, the compensation coil, and the feedback control circuit.

**Figure 2:**
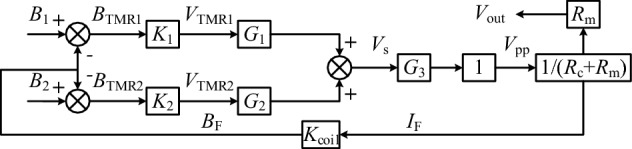
Mathematical model of dual air-gap closed-loop TMR current sensor.

Its working principle is that the wire passes through the designed magnetic core, and two TMR elements are arranged respectively in the centre of the two air gaps. The differential operational amplifier amplifies the output signals of the TMR elements, and the amplified signal drives the triode to generate a feedback current. The feedback coil is wound on the magnetic core, which reduces the magnetic field strength in the air-gap and eventually becomes zero. At this time, the magnetic field generated by the feedback coil and the magnetic field generated by the wire is equal in magnitude and opposite in direction. The measured current can be calculated by measuring the current on the feedback coil through the sampling resistor. The mathematical model is shown in Fig. 2.

For a dual air-gap closed-loop control TMR current sensor, the output voltage of the sensing section is:

1 $${V}_{s}={K}_{1}{G}_{1}{B}_{{\text{TMR}}1}+{K}_{2}{G}_{2}{B}_{\text{TMR2}}.$$

*K*_1_ and *K*_2_ are the sensitivity coefficients of TMR sensor elements 1 and 2, respectively, and *G*_1_ and *G*_2_ are the magnifications of differential amplifiers 1 and 2, respectively. ${B}_{{\mathrm{TMR}}_{1}}$ and ${B}_{{\mathrm{TMR}}_{2}}$ are the magnetic induction intensity in the air-gaps sensed by the two sensor elements. The air-gap magnetic field is the difference between the magnetic field generated by the current to be measured and the magnetic field of the compensation coil:

2 $$\left\{\begin{array}{c}{B}_{{\mathrm{TMR}}_{1}}={{B}_{{\mathrm{gap}}_{1}}=B}_{1}-{B}_{F}\\ {B}_{{\mathrm{TMR}}_{2}}={B}_{{\mathrm{gap}}_{2}}={B}_{2}-{B}_{F}\end{array}\right..$$

*B*_*F*_ is the feedback magnetic induction at the air-gap:

3 $${B}_{F}={K}_{\text{coil}}{I}_{F}.$$

*K*_coil_ is the current-magnetic field conversion coefficient of the compensation coil, which is related to the shape of the coil and the number of turns. The compensation coil current is determined by the power amplifier of the sensor and the resistance value of the compensation coil:

4 $${I}_{F}=\frac{{V}_{\text{pp}}}{{R}_{\text{coil}}+{R}_{m}}.$$

In the equation, *V*_pp_ is the output voltage of the power amplifier, *R*_coil_ is the resistance value of the compensation coil, and *R*_m_ is the resistance value of the sampling resistor. Therefore, the *B*_F_ at both air-gaps is the same.

### Error source analysis

The first source of error is that the performance of TMR components is not entirely consistent due to the manufacturing process, so *K*_1_ and *K*_2_ in Eq. (1) are not the same. For this error, *G*_1_ and *G*_2_ can be adjusted through the circuit to make *K*_1_*G*_1_ = *K*_2_*G*_2_.

The second source of error is the difference between the magnetic flux density ${B}_{{gap}_{1}}$ and ${B}_{{gap}_{2}}$ at the two air-gaps. It can be seen from the above analysis that the *B*_F_ at the two air-gaps is the same, so the magnetic induction intensities *B*_1_ and *B*_2_ generated by the conductors should be studied. When the conductor is at the centre of the coil, according to Ampere’s loop law:

5 $$\oint H\cdot dl={H}_{1}\cdot \left(2\pi r-2d\right)+2{H}_{2}d,$$

where *H*_1_ is the magnetic field strength in the magnetic core, *H*_2_ is the magnetic field strength of the air-gap, *r* is the average radius of the magnetic core, and *d* is the length of a single air-gap. Due to the continuity of the magnetic flux, the magnetic induction strength of the core and the air-gap is the same:

6 $${B}_{1}={B}_{2}=\mu {H}_{1}={\mu }_{0}{H}_{2},$$

where *µ* is the permeability of the magnetic core, and *µ*_0_ is the permeability of the air-gap. Since *µ* > > *µ*_0_, *H*_1_ < < *H*_2_:

7 $${B}_{1}={B}_{2}=\frac{{\mu }_{0}I}{2d}.$$

It can be seen that the magnetic induction intensity generated by the energised wire at the air-gap of the magnetic core is proportional to the current of the wire.

However, since the conductor may not be at the circle’s centre, the magnetic induction intensity generated at the two air-gaps may not be equal. In the case of a single air-gap, only the value of *B*_1_ or *B*_2_ can be measured, resulting in an eccentricity error. But the dual air-gap structure can reduce the eccentricity error by measuring and calculating the arithmetic mean of *B*_1_ and *B*_2_.

The third source of error is that the magnetic field strength changes abruptly at the air-gap, and the magnetic field distribution in the air-gap is non-uniform due to the influence of the leakage flux. In this case, if the TMR element is not strictly placed in the centre of the air-gap, even if the wire is located in the centre of the magnetic ring, the outputs of the two TMR sensor elements will be quite different due to the inconsistency of the air-gap magnetic field environment in which they are located. The higher sensitivity of the TMR element, the larger the amplified of this error. In this case, in Eq. (2), ${B}_{{\mathrm{TMR}}_{1}}$ is not equal to ${B}_{{\mathrm{gap}}_{1}}$ and ${B}_{{\mathrm{TMR}}_{2}}$ is not equal to ${B}_{{\mathrm{gap}}_{2}}$, which is a hindrance to achieving high-precision measurement.

### Magnetic field uniformity coefficient

It can be seen from the above analysis that optimising the magnetic circuit to minimise the measurement error caused by the eccentric position of the TMR sensing element is the critical issue in improving the measurement accuracy of the sensor, and the position error of the TMR element is related to the inhomogeneity of the magnetic field distribution. Therefore, it is possible to optimise the magnetic circuit by reasonably designing the geometric parameters of the magnetic core to reduce the measurement error. In this regard, the concept of the magnetic field uniformity coefficient of the dual air-gap TMR current sensor is introduced, and the magnetic field uniformity coefficient λ is defined as:

8 $$\lambda =\frac{{\left\{\left|{B}_{0}-{B}_{min}\right|,\left|{B}_{0}-{B}_{max}\right|\right\}}_{max}}{{B}_{0}}.$$

*B*_0_, *B*_min_ and *B*_max_ are the magnetic induction intensity of the centre of the observation region in the air-gap, the minimum and maximum values of the magnetic induction intensity of the observation region, respectively. The closer the magnetic field uniformity coefficient is to 0, the more uniform the magnetic field distribution in the region is, and the smaller the measurement error is. Therefore, with the minimum magnetic field uniformity coefficient as the target, the geometric parameters of the core of the compensation coil are optimally designed so that the magnetic field of each air-gap has good uniformity, which can ensure the consistency of the magnetic field environment in which multiple sensing elements are located.

## Parameter optimisation based on the minimum magnetic field uniformity coefficient

It is difficult to use theoretical mathematical equations to calculate the magnetic field uniformity coefficients for different parameters, so finite element simulation is used to simulate the magnetic field uniformity coefficients to optimise the design core parameters. The simulation model is established in Maxwell finite element simulation software.

### Selection of the magnetic core material and shape

**Figure 3:**
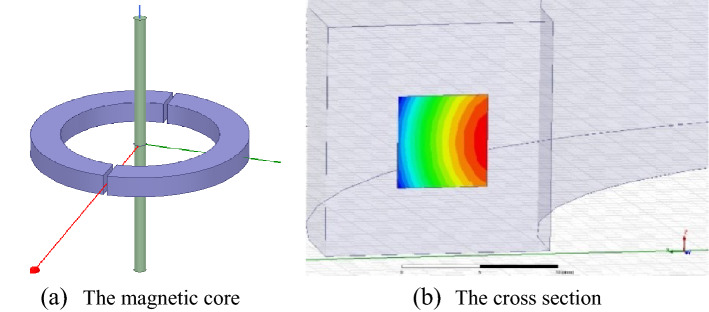
The established finite element simulation model.

The finite element simulation model of the magnetic core and its cross section established in this paper is shown in the Fig. 3.

**Figure 4:**
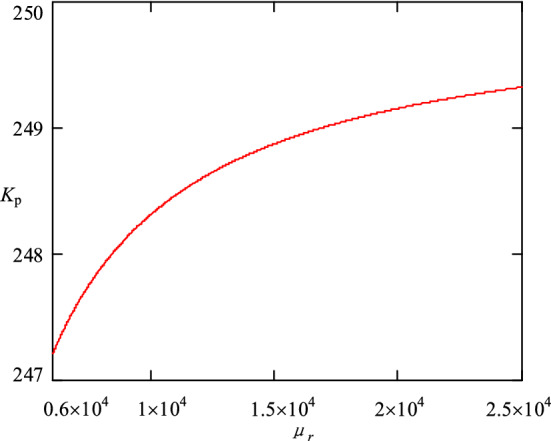
The variation curve of *K*_*p*_.

The magnetic core is mainly used to collect magnetic field, improve magnetic field strength, and thus improve sensitivity and electromagnetic shielding. The key parameter of magnetic core is permeability *μ*_*r*_. It means the ability of the magnetic core to gather magnetic lines. From the perspective of transfer function, high permeability can make the compensation magnetic field coefficient *K*_*p*_ larger. When the size of the magnetic ring and the length of the air gap are determined, the change curve of *K*_*p*_ is shown in Fig. 4. As can be seen, when *μ*_*r*_ increases to a certain extent, *K*_*p*_ does not change significantly. Therefore, it is enough if *μ*_*r*_ can reach 2 × 10^4^.

In addition, coercivity, magnetostrictive properties, temperature stability, etc. shall be considered. Therefore, permalloy is selected as the magnetic core material, its magnetostrictive effect and anisotropy are at a low level, and the initial permeability can reach 2 × 10^4^, the coercivity is less than 2.4A/m, and the resistivity is high, which can reduce the eddy current loss of the magnetic core.

The shape of the magnetic core mainly includes square, round, polygon, etc. The square and polygon structures are mainly used in high current scenes such as bus bars, and the polygon processing is complex. In this paper, the ± 50 A rated current measuring sensor is to be developed, which belongs to medium current. Therefore, the shape of the magnetic ring is designed as a circle, and its section is designed as a square.

### Optimisation of the magnetic core inner radius

The parameters of the magnetic core that need to be optimised include the magnetic core inner radius *r*, the air-gap length *δ* and side length *l* of the magnetic core cross-section. According to the method proposed in this paper, the magnetic field uniformity coefficient of the central region of the air gap where the TMR element is located is calculated as an optimisation index.

**Figure 5:**
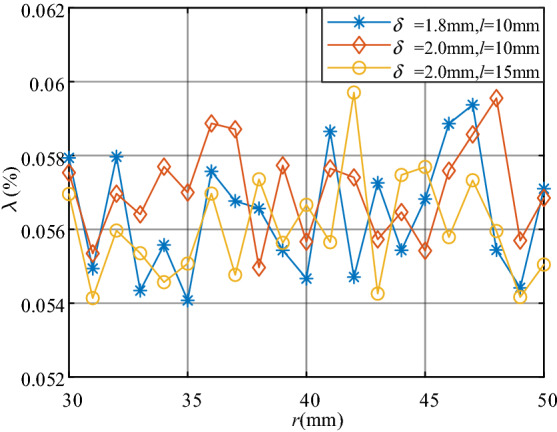
The variation curve of λ for three different group parameters.

First, fix other parameters to analyse the inner radius *r*. Magnetic field analyses are separately carried out from three group parameters: *δ* = 1.8 mm, *l* = 10 mm; *δ* = 2 mm, *l* = 10 mm; *δ* = 2 mm, *l* = 15 mm. And *r* varies from 30 to 50 mm as scan parameter with 1 mm/step. Figure 5 shows the variation curves of *λ* with a variety of *l* for three group parameters.

*λ* is small with no significant change and varies when *l* varies. According to practical application needs, the inner radius of magnetic core *r* = 40 mm is selected.

### Optimisation of the air-gap length and side length

**Figure 6:**
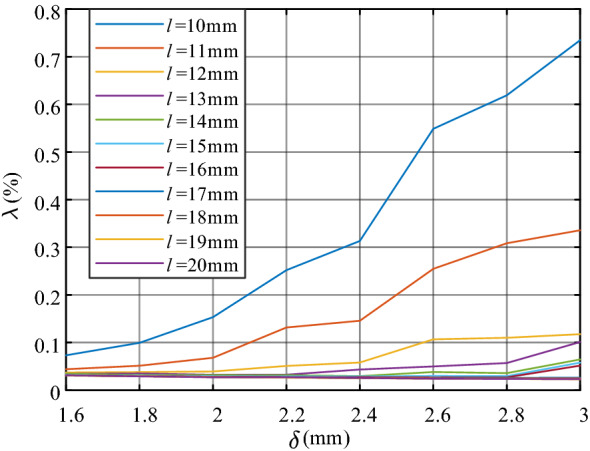
The variation curve of *λ* for different *δ* and *l*.

Considering the element size and its possible position offset, the air-gap centre of 6 mm × 6 mm is set as the placement area to retain a certain margin. This means the minimum side length of the air gap. Set *r* = 40 mm, because the side length of the cross-section of the magnetic core needs to be greater than one-fourth of the inner radius^17^, *l* varies from 10 to 20 mm with 1 mm/step. Considering the thickness of the sensing element, *δ* varies from 1.8 mm to 3 mm with 0.2 mm/step. The variation curves of *λ* can be seen in Fig. 6.

As can be seen, *λ* decreases with the increase of the side length *l*. *λ* increases with the increase of air-gap length *δ* when *l* remains unchanged, but *λ* is always less than 0.1% when *l* > 14 mm, which means that the air-gap magnetic field is evenly distributed. It can be seen from the figure above that the larger the air gap side length is, and the smaller the air gap length is, the better the metering performance is. The air-gap will improve the linearity of the magnetic core and decrease the remanence. However, if the air-gap is too large, the effective permeability of the compensation magnetic core will be reduced. if the air-gap is too large, the effective permeability of the compensation magnetic core will be reduced. Besides, the larger the cross-section of the magnetic core, the overall volume of the compensation coil will increase, and more enameled wires need to be used when winding the coil, which will increase the coil resistance and increase the loss. Therefore, the side length *l* of the air-gap section should not be too large.

**Figure 7:**
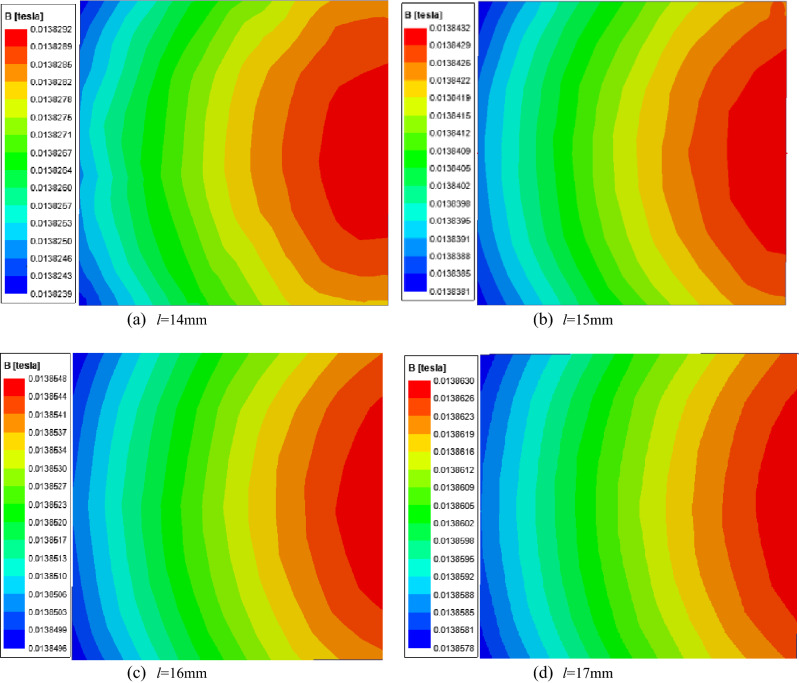
Magnetic field of different *l* when *δ* = 2.2 mm.

Figure 7 shows the magnetic field distribution of different *l* when *δ* = 2.2 mm.

According to simulation and analysis, the geometric parameters of the magnetic core are selected as *r* = 40 mm, *δ* = 2.2 mm, and *l* = 15 mm. In this case, the magnetic field environment where the two sensing elements are located will have a good consistency to reduce the susceptibility of chip space positional deviation.

## Experiment results

According to the above structure and optimisation parameters, a current sensor prototype with a rated transformation ratio of 50A/2 V was fabricated, attaining a sensitivity of 4.006 mV/A, featuring non-intrusive, galvanic isolated contactless current measurement based on the closed-loop TMR technology. The peak current measurement range is ± 75 A, corresponding to 3 V output. And the TMR elements are the TMR2505 produced by MultiDimension Technology Co., LTD. It is a linear magnetic field sensing element with z-axis induction, which has high sensitivity and excellent temperature stability.

### Experimental setup

**Figure 8:**
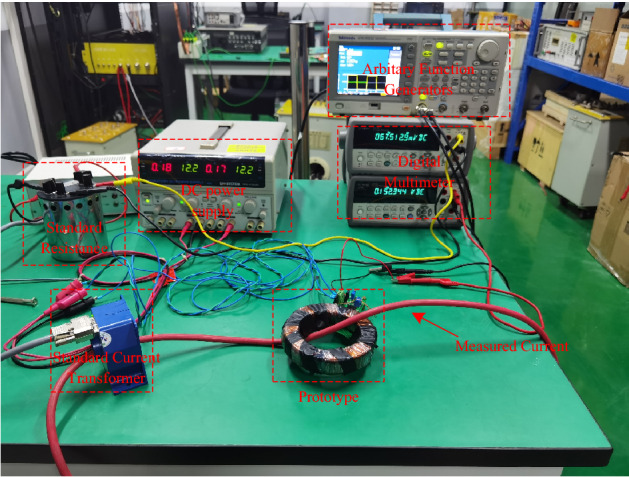
Experimental platform.

A direct comparison methodology was adopted as the current sensor test scheme, which is more mature than the indirect method at present^18^. The principle measures the ratio difference between the current sensor prototype and a high accuracy current transducer selected as the standard current transformer. The experimental platform was constructed as shown in Fig. 8. (Supplementary Table 1).

### Current measurement accuracy experiment

**Figure 9:**
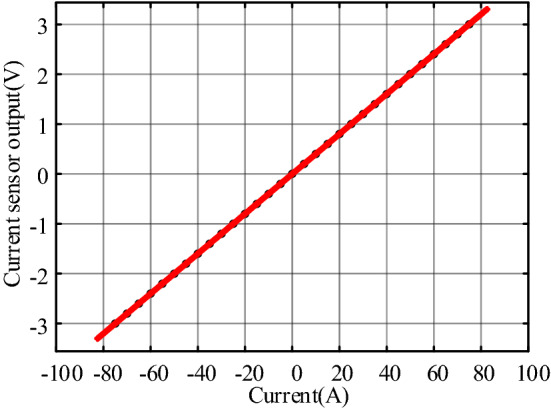
*I*–*V* characteristic of the current sensor.

To acquire the *I*–*V* characteristic of the current sensor, the experiment was carried out as follows. When the primary current is zero, recording the residual output voltage, is the offset voltage *V*_0_ of 2.436 mV. Then the primary current is progressively increased from − *I*_PM_ to − *I*_PM_ (equally spaced *I*_PN_/10 steps). The dates were processed with the least-square fitting method. Figure 9 shows the *I*–*V* characteristic of the current sensor.

The equation of the linear regression line is as follows:

9 $$V=0.04006I+0.003159.$$

The sensitivity of the current sensor is defined as the slope of the linear regression line, it is 0.004006 V/A. To measure linearity, the primary current (DC) is cycled from 0 to *I*_PM_, then to − *I*_PM_ and back to 0 (equally spaced *I*_PM_/10 steps). The linearity error *ε*_L_ is the maximum positive or negative difference ∆*L*_max_ between the measured points and the linear regression line, expressed in % of the rated measured output voltage value *V*_FS_.

**Figure 10:**
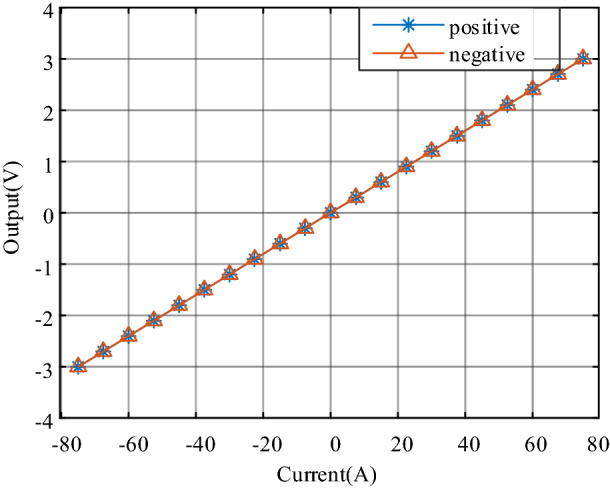
Performance characteristics of linearity.

Figure 10 demonstrates the performance characteristics of linearity. The forward and reverse process fitting curves have good coincidence, and no noticeable return difference is formed. The linear error can be calculated by the following formula.

10 $${\varepsilon }_{L}=\pm \frac{\Delta {L}_{max}}{{V}_{N}-{V}_{0}}\times 100\mathrm{\%},$$

where *V*_N_ is the average absolute value of output voltage when the measured current *I* reach *I*_PN_ and − *I*_PN_. By calculating, the linearity error *ε*_L_ is less than 0.03%. This reflects that the measurement performance of the prototype is little affected by the hysteresis effect.

In addition, the temperature performance of TMR small current sensor is tested. The set ambient temperature change range is − 10 °C to 60 °C, and the temperature change value each time is 10 °C. The result shows that the temperature coefficient of sensitivity (TCS) of the sensor is 422.1 ppm/°C, which meets the measurement requirements.

### Conductor eccentricity error experiment

**Figure 11:**
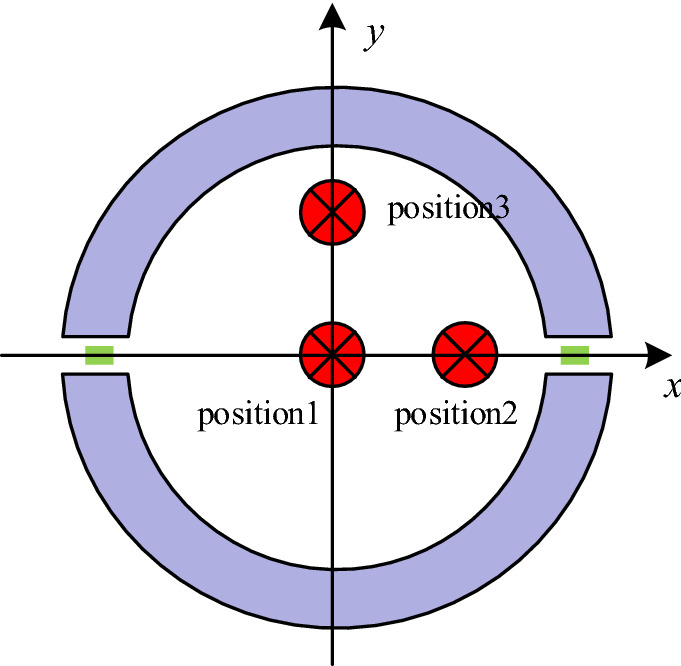
Diagram of three positions of electrical wine location.

To obtain the performance of the dual air-gap current sensor in the spatial deviation of electric wire, comparative experiments were carried out. The schematic diagram of the wire location is shown in Fig. 11. Position 1 is the centre of the magnetic core, as the normal position. The distance between position 2 or position 3 and the centre of the magnetic core is 15 mm, and position 2 and position 3 are close to the air-gap and magnetic core respectively.

**Figure 12:**
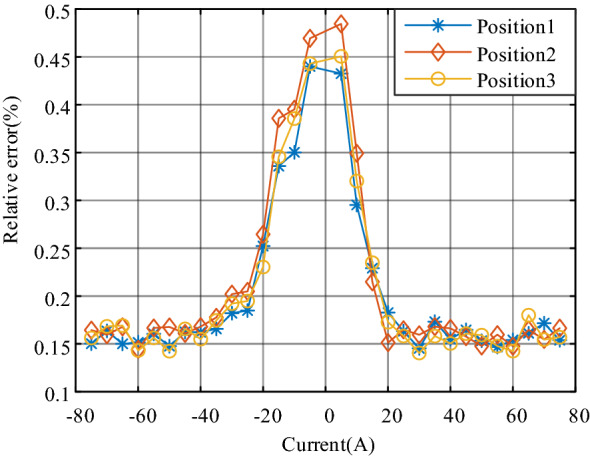
Relative accuracy of spatial deviation.

The relative accuracy of the current sensor in spatial deviation is shown in Fig. 12.

In the whole measurement range, the relative accuracy is less than 0.44%, and the variation range in the standard position is 0.14–0.44%. It decreases as the current increases and keeps stable when the absolute current *I* is more than 40% of *I*_PN_. The performance of the other positions corresponds with that of position 1. The slight difference appears only when the current is small. For example, the maximum relative error is 0.48% at position 2 while it is 0.45% at position 3, but both are highly close to 0.44%. The experiments indicated that the optimal design of the current sensor could decrease the susceptibility of the spatial deviation effect significantly. Furthermore, in the measurement of positive and negative current, the relative error has good symmetry, which means the current sensor can suppress the nonlinearity caused by hysteresis.

It can also be seen from the results that when the measured current is rated at 50 A, the relative error is only 0.15%. When the measured current exceeds the rated value, the measurement error remains unchanged. According to the regulations of relevant standards, the developed sensor prototype meets the requirements of measurement accuracy of 0.2-level accuracy.

## Discussion

To improve the measurement accuracy of the TMR sensor, this paper proposes the concept of magnetic field uniformity coefficient based on analysing the error source and proposes a magnetic circuit optimisation design method based on the minimum magnetic field uniformity coefficient. Based on the optimised design, a sensor with a rated measurement current of ± 50 A and a peak measurement current of ± 75 A is developed. The linearity error is less than 0.03%, and the relative accuracy achieved is less than 0.2% at primary nominal current 50 A and is less than 0.44% over the entire measurement range from − 75 to 75 A when the electric wire is at the normal position. And the test results show that the prototype performance is not affected by the wire position or the current direction. The error of the current measurement is effectively reduced.

## Supplementary Information

Supplementary Information.

## Data Availability

The data that support the findings of this study are available from the corresponding author upon reasonable request.
