# Supplementary material for: Optimal design of dual air-gap closed-loop TMR current sensor based on minimum magnetic field uniformity coefficient
Source: Sci Rep. 2023 Jan 5;13:239. doi: 10.1038/s41598-022-26971-9 (PMC9816158; doi:10.1038/s41598-022-26971-9)
Supplement: Supplementary file 1 — Supplementary Information. [file 41598_2022_26971_MOESM1_ESM.docx]

### Experimental Setup

Compensation current of compensation coil is sampled by the 20Ω high power thin film wraparound chip resistance of VISHAY. Direct comparison methodology was adopted as the current sensor test scheme, which is more mature than indirect method at present. The principle is to measure the ratio difference between the current sensor prototype and a high accuracy current transducer selected as standard current transformer. The diagram of current sensor test scheme is illustrated in Figure 1.

**Figure 1.** Diagram of direct comparison methodology

Table 1 shows the specifications of the devices in the test platform. The laboratory prototype is powered by DC power supply GPS-4303C. The standard current transformer is IT 65-S with a typical accuracy of ±0.03% made by LEM Electronics(China) Co.,Ltd., which meets the requirements of current sensor testing.

**Table 1.** Specification of the devices in the test platform

| Device | Model | Manufacturer | Features |
| --- | --- | --- | --- |
| DC power supply | GPS-4303C | Guwei Electronics Co., Ltd. | -- |
| Digital Multimeter | Agilent 34410A | KEYSIGHT Technologies Co.,Ltd. | DCV 30ppm |
| Standard Current Transformer | IT 65-S | LEM Electronics (China) Co., Ltd. | 0.03% |
| DC Standard Resistance | BZ3-10Ω | Shanghai Precision Instrument Co.,Ltd. | 0.01% |
